# COGNITIVE FRAILTY IN RURAL KOREAN OLDER ADULTS: A GENDER-BASED EXAMINATION

**DOI:** 10.1093/geroni/igae098.2480

**Published:** 2024-12-31

**Authors:** Ah Ram Jang, Hae Sagong

**Affiliations:** Yeoju Institute of Technology, Yeoju, Kyonggi-do, Republic of Korea; Auburn University, Auburn, Alabama, United States

## Abstract

Cognitive frailty, characterized by the simultaneous presence of physical frailty and cognitive impairment, is strongly associated with dementia and mortality, with prevalence among community-dwelling older adults ranging from 0.9% to 12.0%. Rural residents exhibit a higher risk, underlining vulnerability to health issues. However, there is a significant research gap regarding factors influencing cognitive frailty, especially in rural areas. Our cross-sectional survey conducted from May to September 2020 in rural areas of South Korea aimed to address this gap. Participants (n=376) aged 65 or older were recruited from 60 primary health care posts. Incomplete data excluded 32 participants from analysis. Cardiovascular Health Study frailty index and Korean Mini-Mental State Examination were used to categorize cognitive frailty.T-tests, Chi-squared tests, and logistic regression analysis were employed for statistical analysis. The results indicate that among males, higher nutritional scores were associated with a reduced likelihood of cognitive frailty. Conversely, in females, older age heightened the risk. Further, factors such as education, higher nutritional scores, and active social participation were linked to a decreased likelihood of cognitive frailty in females. Recognizing these gender-specific influences, tailored interventions are paramount. Strategies should aim at enhancing nutritional status for both genders while placing a specific emphasis on promoting increased social participation among females. This study enhances our understanding of cognitive frailty in rural older adults and provides valuable insights for the development of targeted interventions.

